# Conversation analysis, psychopathology and subjective experience in patients with schizophrenia

**DOI:** 10.1192/j.eurpsy.2021.439

**Published:** 2021-08-13

**Authors:** V. Lucarini, F. Cangemi, F. Paraboschi, J. Lucchese, B. Daniel, C. Marchesi, M. Tonna

**Affiliations:** 1 Department Of Mental Health, Local Health Service, Parma, Italy; 2 Ifl-phonetics, University of Cologne, Cologne, Germany; 3 Department Of Neuroscience, University of Parma, Parma, Italy

**Keywords:** conversation, psychopathology, self disorders, schizophrénia

## Abstract

**Introduction:**

Patients with schizophrenia show severe difficulties in interpersonal communication, including impairments in conversation skills, like the turn-taking. To our knowledge, very few studies to date have taken into account conversation analysis in order to investigate turn-taking in schizophrenia patients.

**Objectives:**

To investigate the conversational patterns in schizophrenia patients; to assess possible associations between dialogic features, abnormal subjective experiences and symptom dimensions.

**Methods:**

Thirty-six patients with Schizophrenia underwent an interview, subsequently analyzed with an innovative semi-automatic analysis. Positive and Negative Syndrome Scale (PANSS) was adopted for the investigation of psychopathology and Examination of Anomalous Self Experience (EASE) for Self-Disorders.

**Results:**

Dialogic exchanges are graphically represented in Figure 1. An inverse correlation was found between participant speaking time and PANSS negative symptoms score (r = -0.44, p value < 0.05; Figure 2), whereas no associations were found between conversational variables and PANSS positive or disorganization dimensions. Finally, a positive correlation was found between the EASE item “spazialization of thought” and average pause duration (r = 0.42, p value < 0.05).

**Figure:**
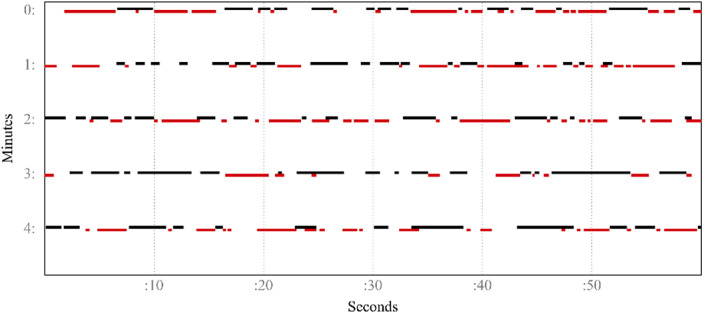

**Figure:**
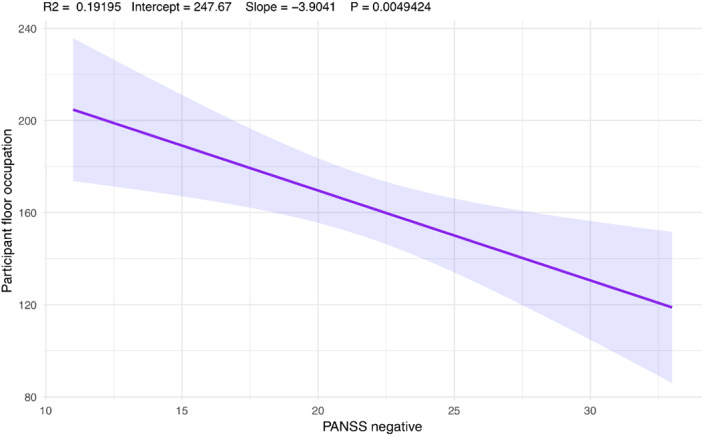

**Conclusions:**

The finding of a relationship between negative symptoms and conversational patterns suggest that conversational features in schizophrenia are expression of the “core” negative dimension of the disorder. The association with the phenomenon of thought spatialization seems to suggest that the disturbances of the stream of consciousness impact on natural dialogic interactions. Ultimately, conversation analysis seems a promising tool to study dialogic exchanges of patients with schizophrenia.

**Disclosure:**

No significant relationships.

